# Identification and analysis correlation between hub genes and immune cell infiltration related to LPS-induced cognitive impairment

**DOI:** 10.1016/j.heliyon.2024.e37101

**Published:** 2024-08-31

**Authors:** Wang Qiang, Wen Juan Deng, Shu Ling Song, Ling Hui Pan

**Affiliations:** a Department of Anesthesiology, Guangxi Medical University Cancer Hospital, Guangxi, China; b Department of Radiology, Guangxi Medical University Cancer Hospital, Guangxi, China; c The Fourth People's Hospital of Nanning, Guangxi, China; d Guangxi Clinical Research Center for Anesthesiology, Guangxi, China; e Guangxi Engineering Research Center for Tissue & Organ Injury and Repair Medicine, Guangxi, China; f Guangxi Health Commission Key Laboratory of Basic Science and Prevention of Perioperative Organ Disfunction, Guangxi, China

**Keywords:** Cognitive impairment, Gene expression Omnibus (GEO), hub genes, Functional enrichment analysis, Protein-protein interaction (PPI), CIBERSORT X

## Abstract

**Background:**

The occurrence of immunity and inflammation outside the central nervous system frequently results in acute cognitive impairment among elderly patients. However, there is currently a lack of standardized methods for diagnosing acute cognitive impairment. The objective of our study was to identify potential mRNA biomarkers and investigate the pathogenesis of acute cognitive impairment in mice brains.

**Methods:**

To analyze changes in hub genes associated with acute cognitive impairment, bioinformatics analysis was performed on the mouse brain injury data of sterile saline control group and lipopolysaccharide (LPS) induced experimental group in Gene Expression Omnibus (GEO). Functional analysis was conducted using Gene Ontology (GO), Kyoto Encyclopedia of Genes and Genomes (KEGG), which facilitated to identify some potential mRNA biomarkers for hub gene expression in mice brains. Additionally, the "CIBERSORT X″ R kit was employed to examine immune cell infiltrations of mice brains in LPS group and saline group.

**Results:**

In the LPS and saline group, 102 significantly upregulated differentially expressed genes (DEGs) and 32 downregulated DEGs were identified. The pathway enrichment analysis using GO and KEGG revealed that these DEGs were mainly related to the regulation of cytokine, cytokine-cytokine receptor interaction, as well as protein interaction with cytokine and cytokine receptor. Immune cell infiltration analysis indicated potential involvement of M1 macrophages, NK cells resting, T cells CD4 memory, and T cells CD8 naive in the process of cognitive impairment. By constructing a protein-protein interaction (PPI) network, five hub genes (Cxcl10, Cxcl12, Cxcr3, Gbp2, and Ifih1) showed significant associations with immune cell types by using a threshold Spearman's rank correlation coefficient of R > 0.50 and p < 0.05.

**Conclusion:**

The mRNA expression profile of the mice brain tissues in the LPS group differed from that in the normal saline group. These significantly expressed mRNAs may act an importance in the pathogenesis of acute cognitive impairment through mechanisms involving immunity and neuroinflammation.

## 1 Introduction

Acute cognitive decline is a prevalent condition observed in elderly patients visiting the emergency department, often caused by infections outside of the central nervous system (CNS). Peripheral immune system activation triggers microglia in the brain to generate inflammatory cytokines, which are accountable for inducing behavioral impairments. Several literature reviews have extensively explored the association between cognitive decline and changes in immunity [[1], [2], [3]], indicating that there existed a complex interplay between physical activity, cognition, amyloid beta (Aβ) accumulation, and the adaptive immune system in cognitive impairment. The cellular components of the immune system play a significant role in both the onset and development of cognitive decline. Current research has primarily focused on three pathophysiological mechanisms underlying acute cognitive decline [[4], [5], [6]]: acute cognitive decline resulting from cerebral alterations such as brain shrinkage and changes in pathways or axes, neuroinflammation, and modifications to genes within the hippocampus. The precise pathophysiological mechanisms contributing to cognitive impairment remain unclear at present time, thereby limiting available treatment options and imposing substantial burdens on public health and economy.

Over the past few decades, an abundance of biomarkers has been proposed for the detection of cognitive impairment [7,8]. However, so far none has demonstrated adequate sensitivity or specificity to be utilized in clinical practice. In recent years, many studies have found that a variety of genes and cell signaling pathways are involved in the occurrence and development of cognitive decline [9,10], but still do not know exactly how this potential relationship works at present. With advances in high-throughput sequencing technology and molecular biology methods, it may be now feasible to explore disease pathogenesis at a molecular level.

To gain insights into the molecular mechanisms that regulate neuronal activity and establish an objective diagnostic method, our study aimed to discover new indicators of potential molecular processes and predict brain tissue molecular biomarkers in mice with cognitive impairment. Our goal was to employ bioinformatics techniques through the Gene Expression Omnibus (GEO) database to identify key mRNA molecules associated with cognitive impairment and investigate their expression, functionality, and interactions.

## 2 Methods

### 2.1 Microarray data

**Fig. 1:**
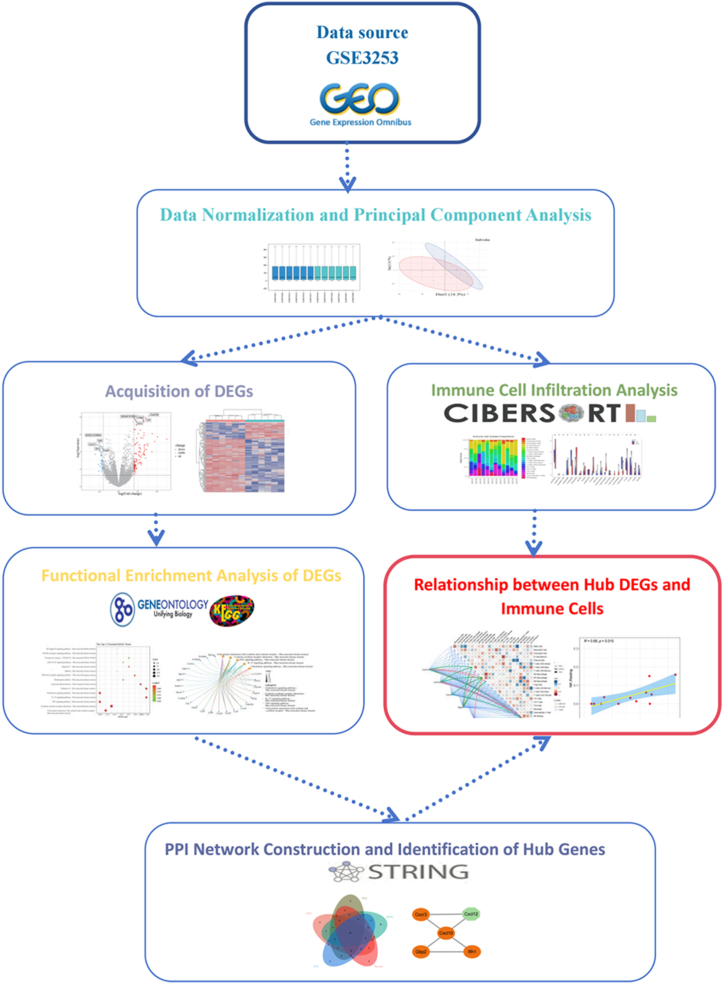
Flow chart of the study.

The microarray dataset GSE3253, submitted by Godbout JP et al., was obtained from GEO database (www.ncbi.nlm.nih.gov/geo/) for the purpose of identifying genes expressed in cognitive impairment samples induced by lipopolysaccharide in mice compared to control brain tissues treated with sterile saline. GEO is an open-access repository for functional genomics data, including high-throughput gene expression data, chips, and microarrays. The study utilized GPL1261 [Affymetrix Mouse Genome 430 2.0 Array] as the platform for conducting expression profiling arrays. A total of 12 samples were analyzed: 6 from mice injected with lipopolysaccharide (LPS group) and 6 from mice injected with sterile saline group (Fig. 1). The raw text file and probe annotation file were acquired and subsequently matched the probes to their corresponding gene symbols based on the platform's annotation information. The entire dataset was made available online without the authors' involvement in conducting any human or animal experiments.

### 2.2 Data processing and identification of DGEs mRNAs

The GSE3253 dataset's series matrix files contained normalized log-expression values, which were useful for conducting differential gene expression analysis. The reproducibility of the data was confirmed by conducting principal component analysis (PCA), and PCA plots were generated using the 'ggord' package in R. Normalization and differential expression analysis of genes in LPS samples and control samples were performed using the ‘limma’ package. Differentially expressed genes (DEGs) were considered significant if they had an adjusted P-value below 0.01 and a |log fold change (FC)| greater than 2. To visualize the DEGs, R was used to create a heatmap and volcano plot.

### 2.3 Functional enrichment analysis

The utilization of Gene Ontology (GO) analysis and Kyoto Encyclopedia of Genes and Genomes (KEGG) Pathway Analysis is widely employed for conducting comprehensive studies on functional enrichment at a large scale. To further investigate potential biological processes (BP), molecular functions (MF), and cellular components (CC), KEGG pathway enrichment analysis is utilized. The 'clusterProfiler' package was utilized to perform GO term and KEGG (www.kegg.jp/kegg/kegg1.html) pathway enrichment analyses using DEGs, with a significance threshold set at 0.05.

### 2.4 Screening of hub genes

To systematically examine the biological functionalities of the DEGs identified between the two groups, we utilized an online search tool called STRING database (STRING, V11.0; https://string-db.org/) to predict the protein functional correlations and protein-protein interactions (PPIs) with a highly reliable filtering condition (score >0.7). We obtained the interaction file (string_interactions.tsv) and used the Perl programming language to derive the network file. Subsequently, Cytoscape software (version 3.9.0; Institute for Systems Biology, Seattle, WA, USA) was employed to assign scores to each gene node based on top five algorithms: Degree, Maximal Clique Centrality (MCC), Density of Maximum Neighborhood Component (DMNC), Maximum Neighborhood Component (MNC), and Edge Percolated Component (EPC). Finally, we determined hub genes by identifying common genes through intersecting them using a Venn diagram.

### 2.5 Immunoinfiltration analysis

The CIBERSORTx algorithm was utilized in this study to estimate the relative proportions of 22 immune cell subtypes based on gene expression levels. The algorithm was configured with a threshold value of 1000. In order to obtain a more comprehensive understanding of immune cell infiltration in both the LPS and control groups, we employed the CIBERSORTx algorithm to accurately ascertain the relative proportions of infiltrating immune cells. Subsequently, we conducted a Wilcoxon test to compare the proportions of infiltrating immune cells between the LPS group and control group, with a significance level set at p < 0.05. The estimated proportions for each type of immune cell were visualized using the 'ggplot2′package. We utilized the Pearson correlation coefficient test to explore the relationship among significant immune infiltrating cells.

### 2.6 Correlation analysis between hub gens and immune cells

The connection between the identified hub genes and levels of infiltrating immune cells was evaluated using Spearman's rank correlation analysis in R software. To visualize these associations, we employed a charting technique with the ‘ggplot2’ package. A significance threshold was set at p < 0.05, along with a minimum Spearman's rank correlation coefficient requirement of R > 0.5.

## 3 Results

### 3.1 Differentially expressed genes

**Fig. 2:**
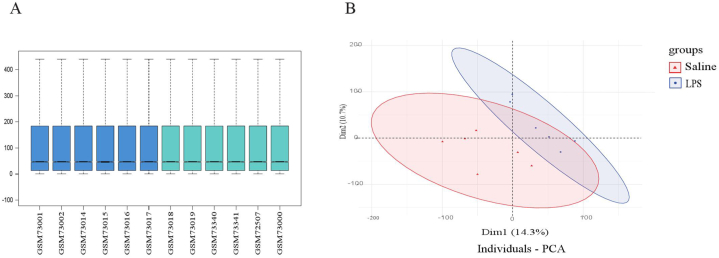
Data normalization and the distribution of differentially expressed genes (DEGs). (A) Box plots illustrated data normalization, the data distributions were neat after background adjustment and normalization. (B) Principal component analysis (PCA), each point in the PCA diagram represented a sample, and the inter-distance between samples reflected the difference. After performing batch correction, individuals with similar genetic backgrounds were effectively clustered together, revealing clear stratification between samples from individuals with cognitive impairment and control tissues.

**Fig. 3:**
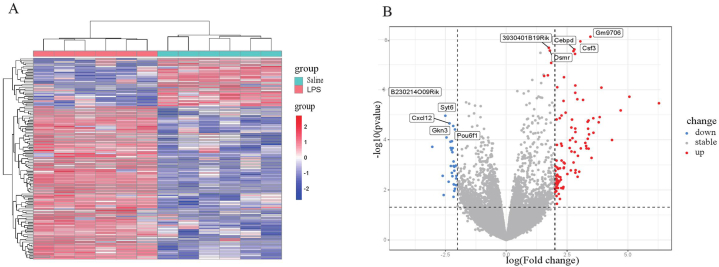
Gene expression profile of GSE3253 is visualized in volcano plots. Differentially expressed genes (DEGs) were marked by colored dots, which represents a DEG met with the criteria of P < 0.05 and |log FC| > 2. Heat map visualization showed alternation for the full range of gene expression patterns between samples with cognitive impairment and control tissues (sterile saline). Purple indicated upregulated genes, and green indicated downregulated genes. The horizontal axis showed clusters of DEGs, and the right vertical axis represented each sample. Gene expression levels were indicated by colors, red (high expression level) and blue (low expression level).

The data consistency of the GSE3253 group was assessed using PCA, which indicated a high level of data reproducibility (Fig. 2A–B). The correlation heatmap of the GSE3253 dataset displayed significant associations among samples in both the LPS group and control group (Fig. 3A). In this investigation, microarray dataset from GSE3253 was identified a total of 21,381 independent genes. Employing the limma package for analyzing differential expression, 134 DEGs between mice induced with LPS and control mice were detected, consisting of 102 upregulated genes and 32 downregulated genes (Fig. 3B).

### 3.2 GO functional and KEGG pathway enrichment analysis of DEGs

**Fig. 4:**
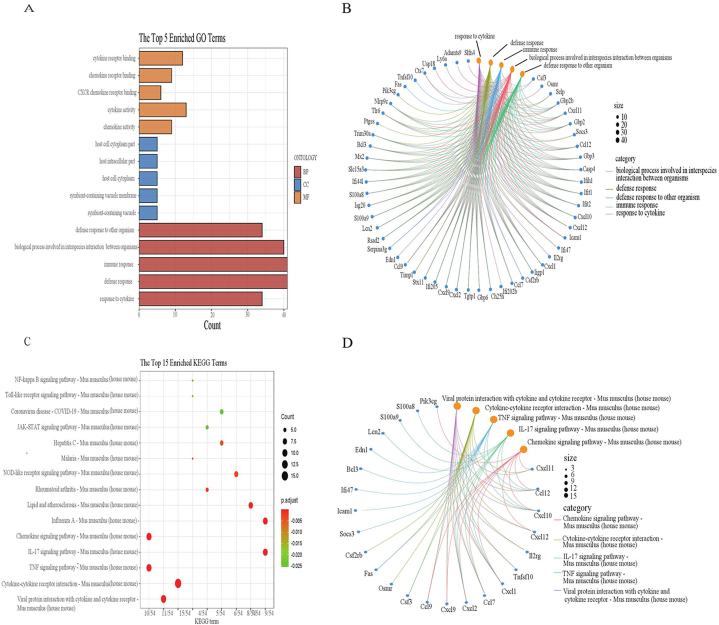
GO and KEGG enrichment analysis. The results of GO were presented by bar plot and circle charts (A and B). The results of KEGG were visualized by bubble and circle graphs (C and D).

The results on GO and KEGG pathway enrichment analyses were showed in Fig. 4A–D. The results from the GO enrichment analysis revealed that BP was predominantly enriched in cytokine, defense response, and immune response. CC showed significant enrichment in symbiont-containing vacuole membrane, host cell cytoplasm, and symbiont-containing vacuole membrane. MF exhibited notable enrichment in chemokine activity, cytokine activity, and CXCR chemokine receptor binding. Furthermore, the KEGG analysis highlighted that the pathways primarily focused on Viral protein interaction with cytokine and cytokine receptor, Cytokine-cytokine receptor interaction, as well as TNF signaling pathway.

### 3.3 PPI network construction and hub gene identification

**Fig. 5:**
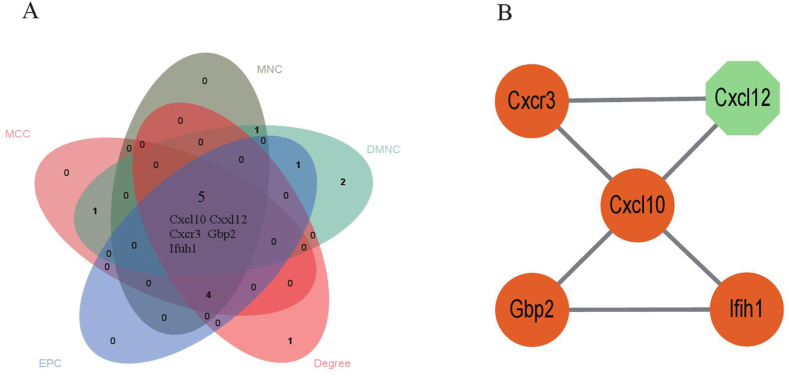
Screening of hub genes. Cytoscape software was employed to assign scores to each gene node based on top five algorithms and hub genes were identified through intersecting of a Venn diagram (A). Hub genes were listed(B). Maximal Clique Centrality = MCC, Density of Maximum Neighborhood Component = DMNC, Maximum Neighborhood Component = MNC, and Edge Percolated Component = EPC.

The PPI network results of DEGs were obtained using the STRING database (Table .1). Subsequently, five different algorithms were utilized to calculate the scores for each gene within the network. Finally, by applying these five algorithms through insertional cytoHubba, we identified the top 10 hub genes (Fig. 5A). The overlapping hub genes from all five algorithms were analyzed using a Venn diagram approach, leading to the identification of five common hub genes: Cxcl10, Gbp2, Cxcl12, Cxcr3, and Ifih1 (Fig. 5B).

**Table 01:** The Top 10 Genes by Cytoscape Software Assign Scores on Top five Algorithms.

method	EPC	Degree	DMNC	MNC	MCC
	Cxcl10	Cxcl10	Usp18	Cxcl1	Cxcl10
	Cxcl1	Cxcl1	Rsad2	Ifit1	Ifit2
	Ifit2	Ifit1	Ccl7	Ifit2	Ifit1
	Ifit1	Ifit2	Cxcl9	Cxcl10	Ifih1
	Ifih1	Gbp3	Cxcl12	Gbp2	Usp18
	Cxcl12	Gbp2	Ifih1	Cxcl12	Gbp3
	Gbp3	Cxcl12	Cxcl2	Cxcr3	Gbp2
	Cxcr3	Cxcr3	Cxcl10	Ifih1	Cxcl1
	Gbp2	Ifih1	Gbp2	Gbp3	Cxcl12
	Cxcl9	Icam1	Cxcr3	Cxcl2	Cxcr3

Note: Edge Percolated Component = EPC, Maximum Neighborhood Component = MNC, Density of Maximum Neighborhood Component = DMNC, Maximal Clique Centrality = MCC.

### 3.4 Immune cells infiltration assessment

**Fig. 6:**
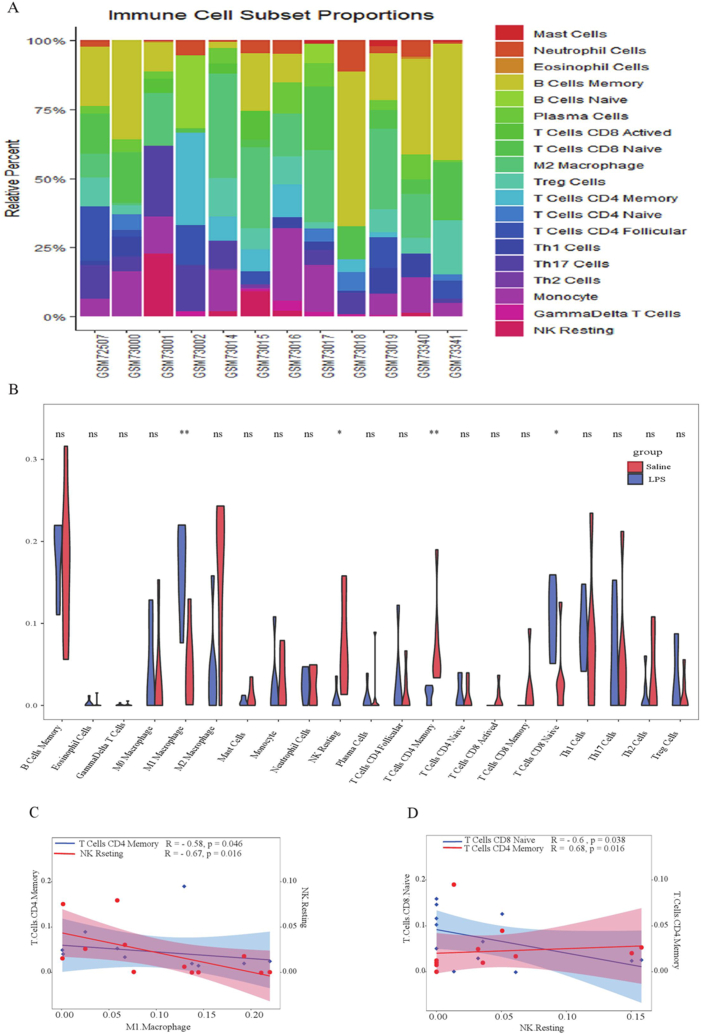
Immune cell infiltration in cognitive impairment and control brain tissues. The composition of 22 kinds of immune cells in each sample was showed in a histogram (A). The 22 types of immune cells between cognitive impairment and control tissues was evaluated, and had 4 significantly differential immune cells (M1 macrophages, NK resting cells, memory CD4 T cells, naive CD8 T cells) (B). The Pearson correlation was calculated among 4 significant immune infiltrating cells (C–D). The color red indicated a positive correlation, while blue represented a negative correlation. The intensity of the color reflected the strength of the correlation.

We compared the disparities in immune cell proportions between LPS mice and the control group through generating a violin plot (Fig. 6A–B). The results of this analysis demonstrated a significant reduction in M1 macrophages, NK resting cells, T cells CD4 memory, and T cells CD8 naive in the LPS group when compared to the control group. Additionally, the correlation analysis revealed that there was a positive association between NK resting cells and T cells CD4 memory; conversely, there was a negative correlation between NK resting and M1 macrophages, also NK resting and T cells CD8 Naive. Moreover, there was a negative correlation between T cells CD4 memory and M1 macrophages (Fig. 6C–D).

### 3.5 Correlation between hub genes and differential immune cells

**Fig. 7:**
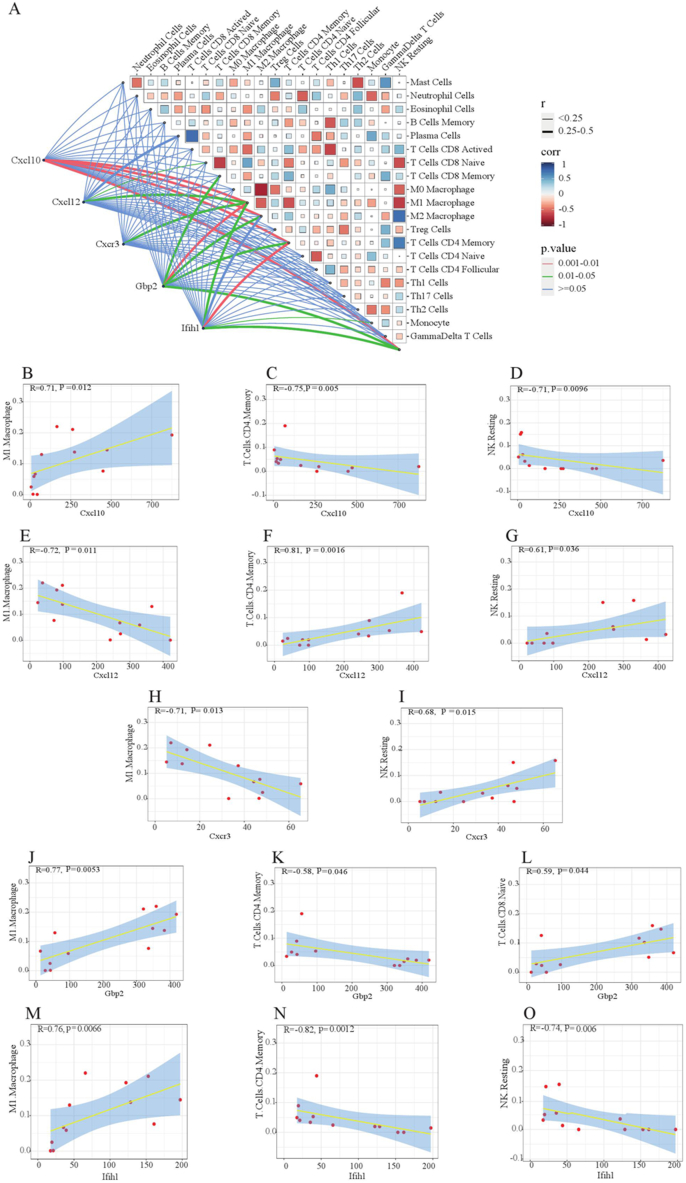
Correlation analysis between hub gens and immune cells. The correlation results were showed in butterfly bat plot(A) and significantly related genes and immune cells by R＞0.5 and P＜0.05(B–O). Red represented a positive correlation, blue represented a negative correlation. The color intensity directly corresponded to the magnitude of the correlation.

The study examined the interaction between five hub genes (Cxcl10, Gbp2, Cxcl12, Cxcr3, Ifih1) and four distinct immune cell types (M1 macrophages, NK resting cells, memory CD4 T cells, naive CD8 T cells) in mice brain tissues affected by cognitive impairment. The correlation results are illustrated in Fig. 7A–O. Key genes and immune cell types that exhibited significant associations were identified using a threshold of R > 0.50 and p < 0.05.

## 4 Discussion

The well-established correlation between various risk factors for cognitive impairment and immune and inflammation-related mechanisms in the CNS is undeniable. Inflammatory elements have the ability to cause inflammation in the CNS by directly crossing the blood-brain barrier (BBB), compromising BBB integrity, or activating multiple signaling pathways [[11], [12], [13]]. The increased brain presence of proinflammatory cytokines leads to excessive activation of microglia and triggers the subsequent release of additional inflammatory mediators within the brain. This inflammation in the CNS further impairs neuronal cells through persistent inflammation, direct effects of inflammatory elements, or indirect influence from non-inflammatory mediators, and ultimately impacts cognitive function and contributes to cognitive impairment. Existing literature analysis revealed a simultaneous upregulation in TNF-α, IL-6, and IL-1β expression in tumor-bearing mice's hippocampus at that same time point, with mRNA levels aligning with observed plasma cytokine increases [12]. These findings suggest that long-term central inflammation resulting from enhanced peripheral innate immunity may be responsible for observed cognitive impairment following surgery among tumor-bearing mice [14].Compared to the control group, the anesthesia group exhibited significantly elevated levels of polarized M1 mRNA expression in macrophages, along with markedly higher mRNA expression levels of TNF-α, monocyte chemoattractant protein 1, and interleukin-6 [15]. The expression of silent information regulator 1(SIRT1) experienced a notable decline subsequent to the induction of postoperative cognitive dysfunction (POCD) induced by cardiac surgery in mice. Administration of SRT1720 effectively suppressed plasma inflammatory cytokine levels and downregulated TLR4 and P65 protein expressions in the hippocampus of POCD mice [16]. Based on these findings, we hypothesize that these abnormal mRNAs may play an essential role in patients suffering from cognitive impairment. Hence, it is imperative to delve into the underlying molecular mechanisms associated with inflammation in the CNS as well as cognitive impairment.

In this study, we initially conducted an independent analysis of genes that were expressed differently in brain tissue samples from mice with LPS-induced cognitive impairment, with the aim of discerning any distinctions between cognitive impairment and control mice. The results revealed a total of 102 up-regulated genes and 32 down-regulated genes that distinguished cognitive impairment mice from their normal counterparts. Subsequently, separate analyses were conducted to ascertain the functions and pathways associated with these differentially expressed genes in both groups. Our findings showed significant enrichment in biological processes related to cytokine response, defense response, immune response, etc., while cellular components exhibited enrichment in symbiont-containing vacuole membrane, host cell cytoplasm, etc. Molecular functions also displayed significant enrichment in chemokine activity, cytokine activity, and CXCR chemokine receptor binding, etc. Furthermore, our analysis using the KEGG database revealed the enrichment of viral protein interaction with cytokines and cytokine receptors, as well as pathways such as Cytokine-cytokine receptor interaction, TNF signaling pathway, IL-17 signaling pathway and Chemokine signaling pathway. Interestingly, previous studies have suggested that alterations in inflammatory processes within the CNS and cellular immunity play a crucial role in cognitive impairment [17,18], with specifically focus on cell-mediated immunity [19]. Based on our analysis results, it is reasonable to speculate that the involvement of the cellular immune system may significantly contribute to the occurrence and progression of cognitive impairment.

By utilizing the PPI network and conducting key module analysis, we have successfully identified five significant genes in mice with cognitive impairment. These genes include Cxcl10, Gbp2, Cxcl12, Cxcr3, and Ifih1. Notably, Cxcl10, Cxcl12, and Cxcr3 belong to the chemokine family - a group of small cytokines or signaling proteins that are secreted by cells [20,21]. Chemokines play a crucial role in immune and inflammatory responses by swiftly being released at sites of inflammation to recruit effector cells into the affected tissue. Specifically, Cxcl10 exhibits potent inhibition of angiogenesis while also significantly impacting thymus function. The upregulation of Cxcl10 expression is observed in spinal cord injury. Although it exerts deleterious effects on cell viability, the presence of Cxcl10 does not hinder the process of scratch healing in HT22 and NSC34 cells [22]. On the other hand, the CXC chemokine receptor 3 (CXCR3), belonging to the G-protein-coupled receptor family, is encompassed within the broader category of CXC chemokine receptors. The expression of this molecule is primarily observed on activated T lymphocytes, NK cells, as well as certain epithelial cells. Extensive researches have demonstrated that interactions between Cxcl10 and its corresponding receptor CXCR3 are pivotal in comprehending various organ-specific autoimmune diseases such as type 1 diabetes, autoimmune thyroiditis, ocular disorders alongside systemic conditions like rheumatoid arthritis, psoriatic arthritis, and systemic lupus erythematosus [[23], [24], [25]]. Cxcl12 (also called as stromal cell derived factor-1 or SDF-1), is widely expressed across different tissues and cell types where it plays an essential role in nerve development, blood vessel formation, hematopoiesis, and immunogenesis. Finally, the GBP2 gene belongs to guanine-binding protein (GBP) family which encodes for interferon-induced proteins responsible for hydrolyzing GTP into GDP. GBP protein regulates inflammasome activation, thereby influencing infection mechanisms caused by diverse pathogens [26].The Ifih1 gene holds immense significance within our bodies due to its implications in autoimmune disorders and viral infections [27].

The distribution of immune cells in the cognitive impairment group and control group was assessed using the CIBERSORT algorithm. Comparison to the control group, there was a significant increase in the proportion of M1 macrophages and CD8 T cells naive within the impairment group, while there was a noticeable decrease in resting NK cells and CD4 memory T cells. The correlation analysis further revealed a negative correlation between Resting NK cells and M1 macrophages (R = −0.67), as well as T cells CD8 Naïve (R = −0.6). Additionally, a positive correlation was observed between Resting NK cells and T cells CD4 memory (R = 0.68). Moreover, there was a negative correlation between T cells CD4 memory and M1 macrophages(R = −0.58). Limited researches have been conducted on immune cell subpopulations in cognitive impairment mice which has led to inconsistent findings [28,29]. The M2 macrophages levels in elderly mice undergoing surgery were lower than that of adult mice surgery and EM-P surgery mice, and the exaggerated cognitive decline and inflammatory response among elderly mice were closely linked with dysfunction of the cholinergic anti-inflammatory pathway [30]. Surgical intervention for tibial fractures triggers the release of mast cells, activation of microglia, and production of inflammatory cytokines, resulting in an acute inflammatory reaction in the brain and subsequent neuronal death, as well as cognitive deterioration. These findings suggest that activation mast cells can initiate microglial activation and cause neuronal damage, thereby contributing to inflammation within the central nervous system (CNS) [31]. The brain tissues of POCD mice were an observed rise in CD4-positive cells concentration along with NK cells in the hippocampus of POCD mice compared to control mice. These findings indicate that peripheral immune cells may contribute to the inflammatory response within the hippocampus following disruption of blood-brain barrier [32].

Given the significant role played by immunoinfiltrating cells and hub genes in cognitive impairment, we further analyzed to explore the relationship between five potential biomarkers (Cxcl10, Cxcl12, Cxcr3, Gbp2, and Ifih1) and immune cell populations that exhibited notable variances in mice with cognitive impairment. The expression of the Cxcl10 gene demonstrated a positive correlation with M1 macrophages, while displaying a negative correlation with T cell CD8 naive and resting NK cells. Conversely, the Cxcl12 gene showed an inverse correlation with M1 macrophages, but displayed a positive association with resting NK cells and T cell CD4 memory. Due to the expression of C-X-C motif chemokine ligand 10 plays a multifaceted role in the pathogenesis and progression of this condition. Furthermore, Cxcl10 antagonists have demonstrated effective suppression of inflammatory immune responses while promoting neuronal regeneration and facilitating functional recovery [33]. The expression of the CXCR3 gene was negatively correlated with M1 giant cells, while positively associated with resting NK cells. The GBP2 gene, on the contrary, exhibited a positive correlation with M1 macrophages and T cell CD8 while demonstrating a negative correlation with T cell CD4 memory. Furthermore, the expression of the IFIH1 gene indicated a positive correlation with M1 Macrophages, but revealed a negative association with resting NK cells and CD4 Memory T cells. In hyperglycemic groups, there was an increase in both transcriptional activity and protein expression levels of markers related to oxidative stress as well as inflammatory cytokines, such as those involved in the Cxcl10/CXCR3 pathway. Long-term evaluation indicated altered dendritic structure within hyperglycemic rats' hippocampus, accompanied by abnormal performance on neurobehavioral tests like Barnes Maze [34]. Cxcl12 triggered the migration of OPCs through the activation of CXCR4-mediated MEK/ERK and PI3K/AKT signaling cascades, ultimately promoting myelin regeneration. The investigation provided evidence that CXCR3 functions as a receptor for chemokines, playing a partial role in facilitating the positive effects of systemic PF4 on the aging brain [35]. The study highlighted the significance of Cxcl12/CXCR4 signaling in spinal dynamics and cognitive flexibility, suggesting that spinal loss could potential for reversibility through targeting RAC1-dependent processes within cortical neurons [36]. The findings of the document suggest that GBP2 gene may potentially play a role in immune function related to the etiology of schizophrenia, which may impact risk assessment, prevention, and treatment strategies [37]. Aicardi-Goutieres syndrome is classified as a monogenic interferon disease resulting from an aberrant mechanism involving intracellular nucleic acid sensing mediated by TREX1, RNASEH2A, RNASEH2B, RNASEH2C, SAMHD1, ADAR1 or IFIH1 [38].

This study has certain limitations that should be acknowledged. Firstly, future research would benefit from a larger sample size and the inclusion of additional datasets to enhance the validity of these findings. Secondly, due to the utilization of publicly available sources for mice data, there is a lack of information regarding age, health status, and personal medication usage. Lastly, it is imperative to conduct further investigate these mRNA discrepancies by developing relevant animal models in order to confirm our results.

## 5 Conclusion

This bioinformatics analysis has successfully identified five valuable molecular targets that warrant further investigation into the mechanisms and selection of biomarkers for cognitive impairment in mice induced by LPS. Several important biological processes and pathways, such as the interaction between cytokines and their receptors, as well as protein interactions involving cytokines and cytokine receptors, may offer novel perspective into the development and progression of cognitive impairment. Furthermore, our findings suggest that factors related to immunity and neuroinflammation play a role in implication of cognitive impairment. To further validate the functionality of these hub genes in relation to cognitive impairment, our team plans to conduct additional molecular biological experiments.

## Funding statement

The authors received no specific funding for this research.

## Institutional review board statement

Not applicable.

## Informed consent statement

Not applicable.

## Data availability statement

Public datasets(GSE3253) were analyzed in this study. The Gene Expression Omnibus database (GEO): https://www.ncbi.nlm.nih.gov/geo/The STRING databasehttps://cn.string-db.org/ CIBERSORTxhttps://cibersortx.stanford.edu/

## CRediT authorship contribution statement

**Wang Qiang:** Data curation, Conceptualization. **Wen Juan Deng:** Writing – original draft, Methodology. **Shu Ling Song:** Methodology, Formal analysis. **Ling Hui Pan:** Writing – review & editing, Conceptualization.

## Declaration of competing interest

The authors declare that they have no known competing financial interests or personal relationships that could have appeared to influence the work reported in this paper.
